# PUBLIC HEALTH PRIORITIES AND FUTURE DIRECTIONS FOR ALZHEIMER'S DISEASE RISK REDUCTION

**DOI:** 10.1093/geroni/igac059.514

**Published:** 2022-12-20

**Authors:** Matthew Baumgart

**Affiliations:** Alzheimer's Association, New York, New York, United States

## Abstract

Reducing risk for diseases and chronic conditions is a fundamental priority of public health. Since 2007, the Alzheimer’s Association has partnered with the CDC on the development of a series of Public Health Road Maps to guide the public health community in addressing cognitive health. In addition, the Alzheimer’s Association’s Public Health Center of Excellence on Dementia Risk Reduction, funded by the CDC, provides guidance on how public health can address the risk factors for cognitive decline and dementia. With recent advancements in the science on dementia risk factors, we can now identify targets for public health action. The addition of a national goal to address dementia risk factors underscores the urgency to act. This presentation will offer a perspective on how public health can move forward, through prioritization and action, in addressing risk factors for cognitive decline and dementia, including by addressing social determinants of health and health equity.

